# Perceptions, Perceived Barriers, and Practices of Physicians’ towards Evidence-Based Medicine

**DOI:** 10.12669/pjms.321.8841

**Published:** 2016

**Authors:** Mukhtiar Baig, Zaid Sayedalamin, Osama Almouteri, Mohammed Algarni, Hassan Allam

**Affiliations:** 1 Mukhtiar Baig, Ph.D., MHPE, Faculty of Medicine, Rabigh, King Abdulaziz University, Jeddah, Kingdom of Saudi Arabia; 2 Zaid Sayedalamin, House officer, King Abdulaziz University Hospital, Jeddah, Kingdom of Saudi Arabia; 3 Osama Almouteri, House officer, King Abdulaziz University Hospital, Jeddah, Kingdom of Saudi Arabia; 4 Mohammed Algarni, House officer, King Abdulaziz University Hospital, Jeddah, Kingdom of Saudi Arabia; 5 Hassan Allam, House officer, King Abdulaziz University Hospital, Jeddah, Kingdom of Saudi Arabia

**Keywords:** Evidence Based Medicine, Physicians, Jeddah, Saudi Arabia

## Abstract

**Objective:**

To investigate physicians’ perceptions and practices towards Evidence-Based Medicine (EBM) and physicians perceived barriers in one institute of Saudi Arabia.

**Methods:**

One hundred seventeen practicing physicians at King Abdulaziz University Hospital, Jeddah were included in the study. A validated questionnaire was used for collecting data. The questionnaire had four parts and included questions addressing perceptions and practices about EBM as well as associated variables and barriers to practicing it.

**Results:**

The majority of the respondents had a positive attitude toward EBM. Only 23.9% of participants reported that they are incorporating EBM into their practice. Knowledge about EBM databases was not good. The most common “regularly” read journal was the New England Journal of Medicine (31.6%), followed by the British Medical Journal (12.0%). Some of the respondents had an understanding and were able to explain to others the technical terms use in EBM such as odds ratio (19.7%), relative risk (22.2%), absolute risk (23.9%) and others. The major perceived barriers to practicing EBM was the lack of free personal time (27.4%), availability and access to information (27.4%), difficulties in involving in whole practice (12.0%) and lack of investment by health authorities (12.8%).

**Conclusion:**

The attitude of the practicing doctors towards EBM was good, but knowledge and practice were not up to the mark.

## INTRODUCTION

Evidence plays most important role in judgment and taking further action. The practice of Evidence-Based Medicine (EBM) keeps a doctor up-to-date about the advanced knowledge and by its application doctor’s clinical performance is increased.^1^

EBM education has been integrated into medical curricula in western countries.^2^ It generally focuses that trainees should be able to utilize “five-step model: ask, access, appraise, apply, and audit, and it emphasizes the translation of the critically appraised evidence into clinical practice”.^2^ Nevertheless, the transfer of evidence into practice is restricted because of several barriers faced by practicing physicians,^3^,^4^ clinical trainers^5^ and trainees.^6^ Several studies are available in the literature, which emphasize the use of EBM in patient care and management and development of EBM guidelines.^1^,^7^

Evidence-based medical practice ensures the correct and best possible patient care according to the international standards. EBM is important for all the practicing doctors in all specialties because it improves diagnosis, clinical judgment, and decision-making.^8^

Generally, physicians treat their patients on the basis of the previous information and understanding they have learned and gained. However, in the medical field, there is exponential increase taking place in the knowledge, and it is a continuous process. Therefore, it has become obligatory for all practicing doctors to have up to date knowledge of new diseases and their management and knowledge of new diagnostic tools and techniques. It is mandatory for the physicians to continuously update his/her knowledge otherwise they will be unable to deliver the patient care services efficiently.^9^

In the present era, the physicians are very busy in the hospitals and their private clinics, and they hardly have sufficient time to read or search some literature related to their field. Therefore, EBM is gaining more importance day by day.

Even though several similar studies have been published from the Kingdom but only a few studies have been published in the last five years. In the present digital era, several EBM resources are available on Smartphone devices, which are commonly used by the doctors, so we assumed that the knowledge and practice of EBM have been improved a lot. Therefore, the present study was designed to investigate current perceptions and practices of physicians’ towards EBM and their perceived barriers in a tertiary care hospital in Jeddah, Saudi Arabia. The results of the present study would help to develop the policy to update practicing doctors’ knowledge and use of EBM in patient care and for overcoming the barriers to practicing EBM.

## METHODS

The present cross-sectional study was conducted among 117 practicing physicians at King Abdulaziz University Hospital, Jeddah, Saudi Arabia, in January -March 2015. The questionnaire was adopted from previously published studies.^10^,^11^ The questionnaire had four parts and included questions addressing perceptions and practices about EBM as well as associated variables and barriers to practicing it. The first part of the questionnaire included data on the personal characteristics of the physicians: age, gender, qualifications, nationality, and years since graduation and practicing experience. The second part addressed practicing characteristics of the physicians: ever-use of resources for clinical decision-making, use of journals and understanding of medical terms. The third part asked about attitudes towards EBM: welcoming current promotion of EBM, colleagues’ positive attitudes towards EBM, whether EBM is useful in daily management, whether EBM improves patient care and if EBM places additional demands on overloaded physicians. The last part included information about barriers to EBM faced by the physicians in obtaining and searching for data. The data was collected by a questionnaire, almost all the participants filled the questionnaire in the presence of the researcher, and the Ethical Approval Committee of the Faculty of Medicine, Rabigh, King Abdulaziz University, Jeddah, approved this study. The verbal informed consent was taken from all participants, and it was assured to all participants that their identity would not be disclosed. The frequency and percentages for categorical data and mean and standard deviation for age were calculated on SPSS 21.

## RESULTS

A total of 117 physicians participated in this study, and the mean age of the respondents was 41.40±11.45 years. In the present study, 47.9% participants were males and 52.1% were females (Table-I). The majority of the respondents had a positive attitude toward EBM, 60.7% were welcoming, and 37.6% were extremely welcoming. The current promotion of EBM was welcomed almost by all the participants (98.3%). Only 23.9% of participants described the incorporation of EBM in their daily practice, 71.8 % mentioned they are doing it sometimes. Only 18.8% of the participants were extremely agreed on the usefulness of research finding in daily patients management (Table-II). Nearly, half of the participants (48.7%) and (47.9%) were aware of the Cochrane Database of Systematic Reviews and Best Evidence Review, respectively while only 8.5% and 7.7% used it, respectively. The most common “regularly” read journal was the New England Journal of Medicine (31.6%), followed by the British Medical Journal (12.0%) while the Middle East Medical Journal (3.4%) and Lancet (3.4%) were the least accessed (results are not shown in the table).

**Table-I:** Characteristics of the participants.

Variables	N	%
*Gender*		
Male	56	47.9
female	61	52.1
*Age*		
25-35 (yrs)	84	71.8
36-45 (yrs)	22	18.8
45-55 (yrs)	6	5.1
55-65 (yrs)	5	4.3
*Working position*		
Consultant	22	18.8
Specialist	23	19.7
Resident	69	59.0
General practitioner	3	2.6
*Year of working in KAUH*		
1-5 yrs	85	72.6
6-10 yrs	18	15.4
11-15 yrs	4	3.4
16-20 yrs	4	3.4
> 21 yrs	6	5.1
*Nationality*		
Saudi	81	69.2
Non-Saudi	36	30.8

N= numbers of respondents, %= percentages, KAUH= King Abdulaziz University Hospital

**Table-II:** Current attitude towards EBM.

Questions	N	%
*Attitude towards the current promotion of EBM*		
Extremely welcoming	44	37.6
Welcoming	71	60.7
Unwelcoming	2	1.7
*Usefulness of research finding in daily patients management*		
Extremely useful	22	18.8
Useful	84	71.8
Unuseful	11	9.4
*General attitude towards EBM*		
Extremely welcoming	57	48.7
Welcoming	46	39.3
Unwelcoming	10	8.5
Extremely unwelcoming	4	3.4
*Practicing of EBM improves patient care*		
Strongly Agree	33	28.2
Agree	69	59.0
Disagree	15	12.8
*EBM used in daily patients management*		
Yes, always	28	23.9
Yes, Most of the time	84	71.8
Yes, sometimes	5	4.3
*Ever use of resources for clinical decision-making*		
Yes, always	10	8.5
Yes, Most of the time	45	38.5
Yes, sometimes	59	50.4
Never	3	2.6
*Awareness of databases relevant to EBM as EBM (from the BMJ publishing group)*		
Unaware	10	8.5
Aware but not used	41	35.0
Read	56	47.9
Used	10	8.5

N= numbers of respondents, %= percentages.

Some of the respondents had a better understanding and were able to explain to others the technical terms use in EBM such as systemic review, odds ratio, relative risk, and absolute risk, (28.2%), (19.7%), (22.2%) and (23.9%), respectively (Table-III). The major perceived barriers to practicing EBM was the lack of free personal time (27.4%), availability and access to information (27.4%), difficulties in involving in whole practice (12.0%) and lack of investment by health authorities (12.8%) (Table-IV).

**Table-III:** Understanding of technical terms used in EBM.

Technical terms	It would not be helpful for me to understand N(%)	Don’t understand but would like to N(%)	Some understanding N(%)	Understand and could Explain to others N(%)
Odds Ratio	5(4.3)	38(32.5)	51(43.6)	23(19.7)
Relative Risk	7(6)	29(24.8)	55(47)	26(22.2)
Absolute Risk	2(1.7)	32(27.4)	55(47)	28(23.9)
Systemic Review	6(5.1)	24(20.5)	54(46.2)	33(28.2)
Meta-Analysis	6(5.1)	28(23.9)	58(49.6)	25(21.4)
Clinical Effectiveness	7(6)	25(21.4)	60(51.3)	25(21.4)
Confidence Interval	8(6.8)	43(36.8)	46(39.3)	20(17.1)
Number needed to treat	7(6)	40(34.2)	51(43.6)	19(16.2)
Heterogeneity	8(6.8)	46(39.3)	46(39.3)	17(14.5)
Publication Bias	10(8.5)	36(30.8)	49(41.9)	22(18.8)

N= numbers of respondents, %= percentages.

**Table-IV:** Perceived major barriers to practicing Evidence-based Medicine in general practice.

Barriers	Frequency	Percent
Lack of personal time	32	27.4
No financial gain in using Evidence-based Medicine	3	2.6
Personal and organizational inertia	13	11.1
Availability and access to information	32	27.4
Lack of investment by health authorities	15	12.8
Lack of hard evidence	6	5.1
Difficulties in involving in whole practice	14	12.0
Too much evidence	2	1.7

Total	117	100.0

## DISCUSSION

Our results found the lack of awareness among practicing physicians about related databases, journals, and review publications. Several previous studies in Saudi Arabia have described similar findings^3^,^11^,^12^ and a study in Qatar also reported similar finding.^8^ Moreover, a study from Pakistan reported that 71% of the physicians and final year students were not aware of the EBM^13^ and a recent study among the dentist reported that only 23% were practicing Evidence- based Dentistry.^14^ Our results show that the lack of awareness among physicians is not changed with the time in spite of easy access to Evidence-Based Medicine literature.

There are several barriers to integration of EBM into clinical practice, which may create a gap between attitude and knowledge of physicians towards EBM. The present study found several perceived barrier to practice EBM and the major barriers were the lack of free personal time, lack of availability and access to information, insufficient investment by health authorities, and unmanageable to involve it in whole practice. Several other studies have reported the physicians’ lack of time is the major barrier to practicing EBM.^10^,^11^ A recent study, reported a lack of EBM skills, lack of time, lack of resources in the hospital to search EBM resources’, were the main perceived barrier.^3^ In KSA and Belgium, lack of EBM skills was the main barrier against practicing EBM followed by lack of time.^3^,^15^ In Jordan, Netherland, and the UK, lack of personal time was the main barrier against practicing EBM.^4^,^10^,^16^ This could be due to high flow of patients in the OPD especially in the government hospitals. However, the medical profession needs a lot of dedication and up to date knowledge to provide the best possible diagnostic and treatment care to the public. Incorrect decisions while treating patients can be very dangerous for patients’ health and life. Therefore, today’s doctor will have to be very careful, precise and accurate for diagnosing and treating the patients. A study suggested that commitment of the hospital administration to patronize EBM practice would be the first step to start implementing EBM in Kingdom hospitals.^3^ It is reported that, “to optimize the transfer of evidence into patient care—through installing EBM education in practice such barriers need to be overcome”.^17^

EBM literature indicates that most physicians’ have a heavy load of patients and therefore, have less time to avail and apply resources of EBM. It is suggested that teaching of EBM skills should be incorporated into the medical curriculum in Saudi Arabia. In the Kingdom, the resources are abundant, and there is need of streamlining of those resources.

In the present survey, less than the quarter participants were extremely agreed on the usefulness of research finding in daily patients management. The welcoming behavior of the respondent was similar to several studies.^7^,^9^,^10^,^18^ Our results indicate that most of the participants had an overall positive attitude towards EBM. These results are similar to another study from KSA,^3^ while few other studies found better positive attitude among physicians around the globe.^7^,^16^

A study in Netherland reported a lack of positive attitude of the participants towards participating in research in general practice, and their knowledge and behavior towards EBM was not up to the mark.^19^ A recent study in India reported lack of awareness among postgraduate students about EBM.^20^

In our study, some of the respondents had an understanding and were able to explain to others the basic terminology used in EBM, such as systemic review, odds ratio, relative risk, and absolute risk and around one-third of the physicians indicated that they would like to understand more. A study in Japanese physicians found less than half of the participants understood the technical terms used in EBM, only 3% were able to explain it to others, and many of the physicians (41%) wanted to understand the terminology more.^7^ In the present study, less than one- aquarter of the respondents stated that they are incorporating EBM into daily practice. Our results are better than a Japanese study that reported only 3% physicians use EBM sources in clinical decision-making.^7^

Recently, a systematic review described that despite having good positive attitudes towards EBM physicians have a lack of knowledge. Furthermore, because of prime importance of EBM appropriate steps needs to be taken for removing the barriers and increasing the knowledge of health care providers.^1^

The attitude of our study participants towards EBM was good, and they perceived it important for improving the quality of their patients care and management. It is also evident from their responses that because of several reasons they are unable to update their knowledge. In the Kingdom of Saudi Arabia, Saudi Commission for Health Specialties (SCFHS) has made Continuing Medical Education (CME) mandatory for every practicing doctor. Every physician has to earn 20-40 CME hours in a year, according to designation but still there is a lot of room for improvement.

The results of the present study regarding the lack of knowledge, and several perceived barriers are similar to several previous studies carried out in the Kingdom,^3^,^11^,^12^ it indicates that in spite of the several steps taken by the SCFHS and easy access to the information and there is some missing link. For improving physicians knowledge and overcoming existing barriers, we have few suggestions.

The certificate of CME should not be given at the end of the lecture, seminar, conference and workshop. There should be an online test after few weeks, and CME certificate should be linked with the passing of the test and for motivating physicians, it would be a good step that authorities should give some exceptional raise in their salaries on a good performance in the test.

A separate and independent CME department should be established to control CME activities, and they should regularly send at least one recent EBM article monthly to the registered physicians according to their specialties. At the end of the year, there should be an online test regarding those articles, which had been sent to them throughout the year, and fifty percent of their CME hours should be linked with physicians success in that test. This step would motivate physicians to study and understand recent advances in the field of medical sciences. Attending lectures or seminars do not have the long-term effects but if they would study and learn themselves and know that they would be assessed then they would try to understand and learn things in a different way.

There is a need to take the initiative to make EBM training mandatory at undergraduate level because today’s doctor is tomorrow’s practicing physician. It is already being taught globally in several medical school curriculums.^21^

### Limitations

The present study has few limitations. First, it is a cross-sectional study and sample is not large enough. Second, all of the statements are physicians self-reported and self-judgment; therefore, the results cannot be generalized to all KAUH doctors. Furthermore, it’s one center study so results cannot be generalized.

## CONCLUSION

Results of our study show that attitude of the practicing doctors towards EBM is good, but knowledge and practice are not up to the mark. There is a need for the intervention to provide better care to the patients’.
